# Network Analyses Based on Machine Learning Methods to Quantify Effects of Peptide–Protein Complexes as Drug Targets Using Cinnamon in Cardiovascular Diseases and Metabolic Syndrome as a Case Study

**DOI:** 10.3389/fgene.2021.816131

**Published:** 2021-12-24

**Authors:** Yingying Wang, Lili Wang, Yinhe Liu, Keshen Li, Honglei Zhao

**Affiliations:** ^1^ Department of Neurology and Stroke Center, The First Affiliated Hospital of Jinan University, Guangzhou, China; ^2^ Clinical Neuroscience Institute, The First Affiliated Hospital of Jinan University, Guangzhou, China; ^3^ Fuwai Hospital Chinese Academy of Medical Sciences, Shenzhen, China

**Keywords:** peptide-protein complexes, network analyses, metabolic syndrome, cardiovascular, cinnamon

## Abstract

Peptide–protein complexes play important roles in multiple diseases such as cardiovascular diseases (CVDs) and metabolic syndrome (MetS). The peptides may be the key molecules in the designing of inhibitors or drug targets. Many Chinese traditional drugs are shown to play various roles in different diseases, and comprehensive analyses should be performed using networks which could offer more information than results generated from a single level. In this study, a network analysis pipeline was designed based on machine learning methods to quantify the effects of peptide–protein complexes as drug targets. Three steps, namely, pathway filter, combined network construction, and biomarker prediction and validation based on peptides, were performed using cinnamon (CA) in CVDs and MetS as a case. Results showed that 17 peptide–protein complexes including six peptides and four proteins were identified as CA targets. The expressions of AKT1, AKT2, and ENOS were tested using qRT-PCR in a mouse model that was constructed. AKT2 was shown to be a CA-indicating biomarker, while E2F1 and ENOS were CA treatment targets. AKT1 was considered a diabetic responsive biomarker because it was down-regulated in diabetic but not related to CA. Taken together, the pipeline could identify new drug targets based on biological function analyses. This may provide a deep understanding of the drugs’ roles in different diseases which may foster the development of peptide–protein complex–based therapeutic approaches.

## Introduction

Peptide–protein complexes are the key components of protein–protein interaction (PPI) networks. Nearly 15–40% PPIs are mediated by these short linear peptides (Neduva et al., 2005). The peptide–protein complexes are proven to play important roles predominantly in both signaling and regulatory pathways, implicating that the peptides are involved in many human diseases (Pawson and Nash, 2003). As a result, the peptides are attracting more attention in drug research fields since they may be the key molecules in the designing of inhibitors or drug targets (Parthasarathi et al., 2008; London et al., 2010).

Due to the characters of peptide–protein interactions, it is reasonable to perform network analyses based on machine learning methods since the relationships between those peptides and proteins could be illustrated clearly in the form of graphs (Zhu et al., 2020; Ji et al., 2021; Yingying et al., 2021). Similarly, some complex diseases are found to be similar based on network analyses, indicating that more relationships between different diseases could be predicted using bioinformatics pipelines (Wang et al., 2019). Many Chinese traditional drugs are shown to play various roles in different diseases, and comprehensive analyses should be performed using networks which may offer more information than results generated from a single level. However, no pipeline aiming to predict the peptide–protein complex as drug targets in different diseases had been proposed. In this study, a network analysis pipeline was designed based on machine learning methods to quantify the effects of peptide–protein complexes as drug targets.

In this pipeline, diseases with at least 20 related genes and drugs with at least one related biological functional term could be used as analysis objects. Diseases that are similar to each other on at least one level (such as medical or biological level) are recommended. The candidate drugs do not need to be proven useful in the diseases analyzed since predicting new roles of the candidate drugs is also one application of the pipeline. Based on the abovementioned concerns, two types of diseases (cardiovascular diseases (CVDs) and metabolic syndrome (MetS)) and a Chinese traditional drug (cinnamon) as a case were chosen.

Cinnamon (*Cinnamomum zeylanicum* and Cinnamon cassia, CA) is one of the most important spices used daily (Hariri and Ghiasvand, 2016). Cinnamaldehyde is one of the main resinous ingredients found in CA, which is commonly used as a Chinese medicine for blood circulation disturbance and inflammation (Sheng et al., 2008; Cao et al., 2010; Yang et al., 2015). It was shown that cinnamaldehyde played important roles in both CVDs and MetS (patients suffering from type 2 diabetes (T2D), and glucose/insulin metabolism disturbance or insulin resistance, and was involved with at least two of the following four items: hypertension, dyslipidemia, obesity, and microalbuminuria defined by the WHO criteria) (Mollazadeh and Hosseinzadeh, 2016). CVDs and MetS are not independent since MetS is one of the most undeniable reasons of CVDs. Besides, there are multiple types of biomarkers identified as common features of CVDs and MetS, such as non-coding RNAs, proteins, and metabolites (Das et al., 2020).

It is of great importance to explore the mechanism of CA since this drug could participate in both of the disease types at the same time (Sheng et al., 2008; Yang et al., 2015)*.* One possible reason may be its antidiabetic action by modulating the insulin and insulin-like growth factor (IGF1) signaling pathways (Schriner et al., 2014) since insulin resistance was proven to play a fundamental key role for MetS complications (Khan et al., 1990). Besides, CA was shown to retard the progression of cardiac hypertrophy and fibrosis *via* blocking the ERK signaling pathway (Zhang et al., 2015; Xiao et al., 2017). However, functional analysis for CA in a system way, especially based on biological pathways, is still lacking.

As an integration of molecular interaction; genetic, cellular, and environmental information processing; and metabolism reactions, biological pathways are often used in systematic analyses of complex diseases such as CVDs, T2D, and cancers (Salt and Hardie, 2017; Kakiuchi-Kiyota et al., 2019; Kaku, 2019). Peptide–protein complexes were also proven to be the key components in pathways. It was postulated that there may be associations between the common pathways shared by CVD/MetS and CA which could be detected based on peptide–protein complex analyses. In this study, a new network analysis pipeline was proposed based on machine learning methods to identify common drug targets in different diseases.

## Materials and Methods

**FIGURE 1:**
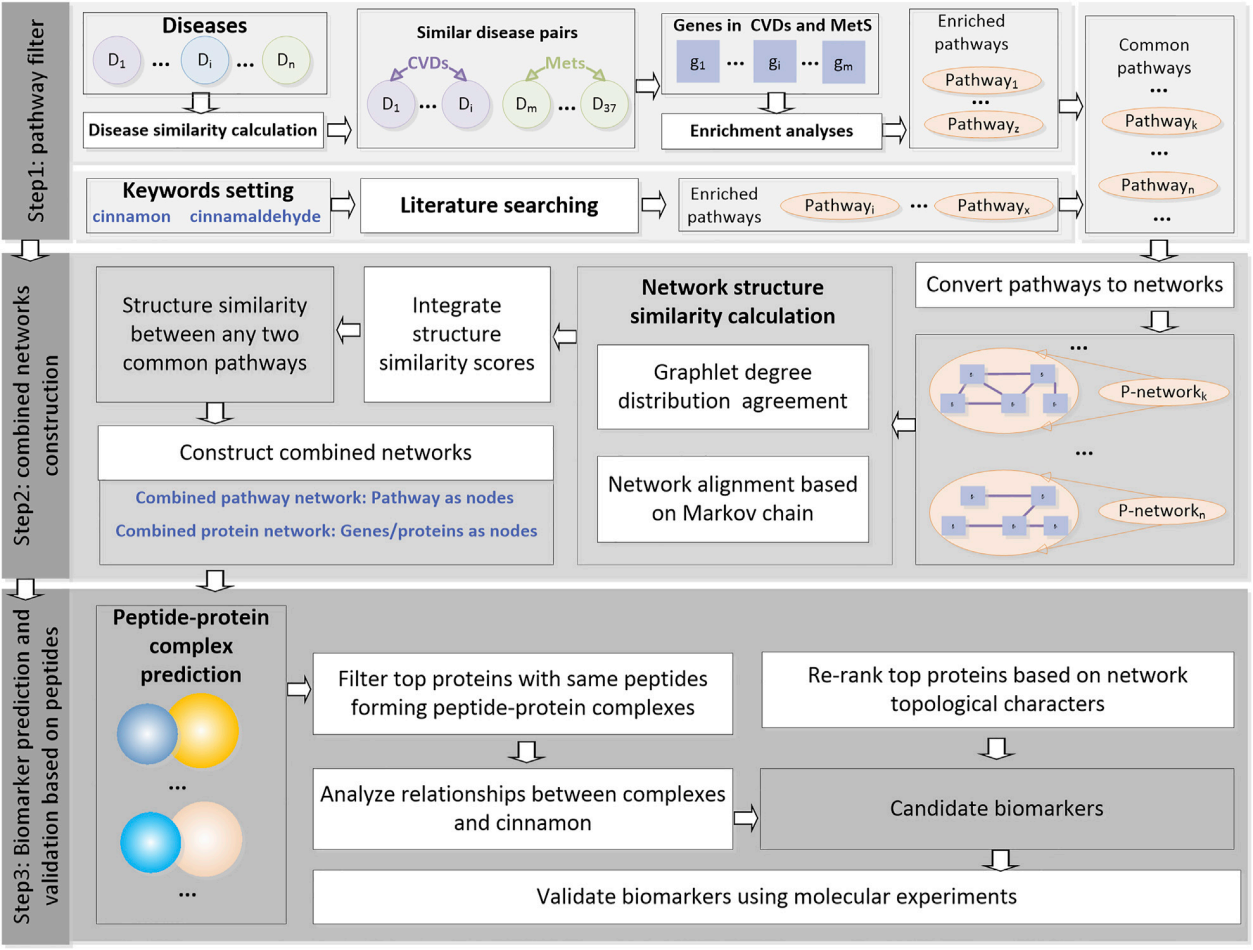
Flowchart of this study. This pipeline consists of three steps, namely, “pathway filter,” “combined network construction,” and “biomarker prediction and validation based on peptides.”

The analyses were performed using the following three steps: (as shown in Figure 1).

Step1: Pathway filter. The similarity between any two selected diseases was calculated and used to filter the disease pairs. Enrichment analyses were performed for the related genes of the disease pairs. Meanwhile, the CA-related pathways were found through literature searching. Common pathways were then filtered and used as the inputs for step 2.

Step2: Combined network construction. All the common pathways were then converted into networks. The network structure similarities were calculated using two types of machine learning methods, and an integrate score was designed to measure the similarity between any two common pathways on the structural level in order to explore the potential correlations of these pathways. The pathways were then merged into a combined pathway network. Proteins in the pathways were merged into a combined protein network.

Step3: Biomarker prediction and validation based on peptides. The nodes in the combined protein network were first ranked according to the network topological characters. Then protein–peptide complexes containing these top proteins as receptors were selected, and the peptides were then clustered. The top genes with peptides clustered into the same clusters were selected as candidate biomarkers and validated using qRT-PCR in a mouse diabetic model that was constructed.

### Disease Similarity Calculation

Methods that can calculate the distances between any two diseases based on any biological or medical level could be used. In this case, a module-based method (Menche et al., 2015) was used*.* The similarity *S*_*ij*_ between two diseases *i* and *j* was calculated as follows:

$$S_{ij}\equiv \langle d_{ij}\rangle -\frac{\langle d_{ii}\rangle +\langle d_{jj}\rangle }{2}.$$

Of which, <*d*_*ii*_ *>* and <*d*_*jj*_ *>*represented the average shortest distances inside diseases *i* and *j*, respectively, while <*d*_*jj*_ *>*represented the pairwise average shortest distance between disease *i* and *j*. The shortest distances were calculated for any protein pairs inside/between diseases using the relationships integrated from multiple molecular interaction levels including protein, regulatory, and metabolic pathways, and kinase substrate.

A z-score was calculated based on the random control networks by 1000 permutations of disease lists preserving randomization. A *p*-value for each *S*_*ij*_ score was calculated using the Mann–Whitney U test. Then FDR was used to obtain the *q*-values.

### Information Converting

#### From Genes to Biological Pathways

The information conversions from genes to biological pathways were performed using the DAVID EASE score (Huang et al., 2009a; Huang et al., 2009b), which was a modified Fisher exact *p*-value. For any disease-related gene list *l*_*i*_ and biological pathway *w*_*a*_, the EASE score was calculated as follows:

$$e\left(l_{i},w_{a}\right)=1-\overset{GH-1}{\underset{i=0}{\sum }}\frac{\left(\begin{array}{c} OH\\ i \end{array}\right)\left(\begin{array}{c} OT-OH\\ GT-i \end{array}\right)}{\left(\begin{array}{c} OT\\ GT-1 \end{array}\right)}.$$

Of which, the calculation methods of GH (gene hits), GT (gene total), OH (genome hits), and OT (genome total) are shown in the following 2*2 table:

**Table:** 

	Number of genes in *l*_*i*_	Number of genes in the genome
Number of genes in *w*_*a*_	GH-1	OH-GH+1
Number of genes not in *w*_*a*_	GT-GH	OT-GT-(OH-GH)

An e-value not above the threshold supported the alternative hypothesis that the probability of the first cell in the 2*2 table was actually greater than that expected under the null hypothesis that the two variables were independent. The conclusion was that there was an association between the row and the column variables in the table, which meant the proportions of those genes falling into each category were different among groups.

### From Biological Pathways to Graphs

The information conversions from biological pathways to protein–protein networks were performed using the R package “graphite” (Sales et al., 2012)*.* The algorithm in this package kept the information of protein complexes, gene families, and removing chemical compounds from the final graphs, which was especially important in the peptide complex analyses of this study.

### Network Structure Similarity Calculation

The network structure similarity calculation algorithms could be divided into two types: alignment-free and alignment-based network comparison (Frigo et al., 2021). In this pipeline, it was recommended to use at least one alignment-free algorithm and one alignment-based algorithm to compare the different networks and combine the scores together.

### Alignment-Free Algorithm Based on Graphlet Degree Distribution Agreement

The alignment-free network comparison algorithms performed the network similarity analyses by quantifying the overall topological similarity between networks, irrespective of node mappings between the networks, and without any conserved edges or subgraph identification. In this pipeline, the algorithm named GDD agreement was chosen, which performed the structural similarity (SS) between networks based on the graphlet degree distribution as follows (Przulj, 2007):

The similarity between any two networks *G* and *W* was calculated as follows:

$$S_{GDD}\left(G,W\right)=\frac{1}{n}\overset{n-1}{\underset{j=0}{\sum }}S_{GDD}^{j}\left(G,W\right).$$

Of which,

SGDDj(G,W)=1−(∑k=1∞[dGj(k)k∑k=1∞dGj(k)k−dWj(k)k∑k=1∞dWj(k)k]2)12.

Of which, $d_{G}^{j}\left(k\right)$ is the sample distribution of the number of nodes in network *G* touching the appropriate graphlet *k* times. The range of *S*_*GDD*_ is [0,1]; a higher score meant the two networks compared were more similar to each other.

### Alignment-Based Algorithm Based on the Hungarian Method

The alignment-based network comparison methods referred to a series of algorithms aiming to find a mapping between the nodes of at least two networks that preserved edges and a large subgraph between the networks. In this pipeline, an alignment-based algorithm was chosen based on a Hungarian method as follows:

The network alignment scores, that is, *S*_*AE*_ (*G, W*) (between any two networks *G* and *W*), were performed using the Hungarian method (Kuhn, 1955) on a square distance matrix *C* (if the sizes of the two networks were different, the larger number of nodes was used), which was calculated as follows:

$$C_{ab}=\sqrt{\underset{t\in T}{\sum }{\left(M_{G_{a,t}}-M_{W_{b,t}}\right)}^{2}}.$$

Of which, $M_{G_{a,t}}=\frac{-\sum_{j=1}^{N_{G}}A_{G_{a,j}}^{\left(t\right)}\ln\left(A_{G_{a,j}}^{\left(t\right)}\right)}{H\left(I^{N_{G}}\right)}$ , where $A_{G_{a,j}}^{\left(t\right)}$ is a transition matrix of network *G*, which was constructed by converting the raw square transition matrix into Markov processes by normalizing each row sum to unity. $A_{G}^{\left(t\right)}$ contained probabilities of edges transferring information from the *i*th to the *j*th member of the system in exactly *t* units of time. For $t\in \left\{2^{y}\right\}\forall y\in \mathbb{N}$ , $t_{\max}\geq 2D$ and $t_{\max-1}<2D$ , where *D* is the max diameter of the two networks *G* and *W* being compared, and the R packages “igraph” and “netcom” were used to perform the calculation.

### Integrated Network Similarity Score

The integrated network similarity scores between the two networks *G* and *W* were calculated as follows:

$$S\left(G,W\right)=S_{GDD}\left(G,W\right)+S_{AE}\left(G,W\right).$$

A higher *S* score indicated that the two networks compared were more similar to each other on the structural level.

### Biomarker Prediction and Validation

The prediction and validation of the biomarkers were performed using the following steps:

1) The proteins in the combined protein network were ranked according to the network topological characters. For each node, degree and node betweenness were calculated. The edge betweenness was calculated for each edge using the R package “igraph.”
2) The protein–peptide complexes containing these top proteins as receptors were selected, and the peptides were then clustered. The high-resolution structures of protein–peptide complexes containing genes in the combined network as receptors were downloaded from the Protein Data Bank (PDB).
3) The top proteins with peptides clustered into the same clusters were selected as candidate biomarkers. The peptide sequences of these complexes were then classified using Hammock (1.2.0) (Krejci et al., 2016), which used hidden Markov model profiles for peptide sequence clustering. The consensus sequence for each cluster was generated using ClustalW (Thompson et al., 1994) and WebLogo (Crooks et al., 2004).
4) The candidate biomarkers were validated using qRT-PCR in a mouse diabetic model constructed as follows:

Fifty-nine male C57 mice (14–16g/28–35 days) were purchased from Guangdong Medical Experimental Animal Center (Certificate No.: 44007200062167, License No.: scxk (Guangdong) 2018-0002, SPF clean grade).

The mice were divided into four groups as follows: 1) Group A (Control + vehicle): 5 mice were given solvent control (0.5% carboxymethyl cellulose solution (CMC)) by gavage; 2) Group B (Control + CA): 6 mice were given CA by gavage (the dose was 20 mg/ kg/ BW); 3) Group C (T2D + vehicle): 24 diabetic mice were given solvent by gavage; 4) Group D (T2D + CA): 24 diabetic mice were given CA by gavage.

Of which, the models of 48 diabetic mice were constructed using streptozotocin (STZ) using the following steps: 1) pretreatment: all the mice were made to starve 12 h before modeling; 2) model construction: STZ was intraperitoneally injected at a dose of 150 mg/ kg/ BW; 3) model test: the blood glucose value was measured continuously after 3 days of STZ injection. If the random blood glucose was >16.7 mmol/ L, the model was considered successful. Otherwise, another injection of STZ was administered until the random blood glucose was >16.7 mmol/ L.

Drug treatment (Groups C and D) was started 5 weeks after modeling. After 7 weeks of administration, all animals were killed, and the hearts of mice were treated with TRIzol and stored at −80°C. Then qRT-PCR was performed for the candidate genes (the top proteins were mapped to their coding genes). The animal experiment was approved and recognized by the experimental Animal Ethics Committee of Shenzhen Sun Yat sen Cardiovascular Hospital (Approval No.: rye2019102806).

## Results

### Pathway Filtrations

**FIGURE 2:**
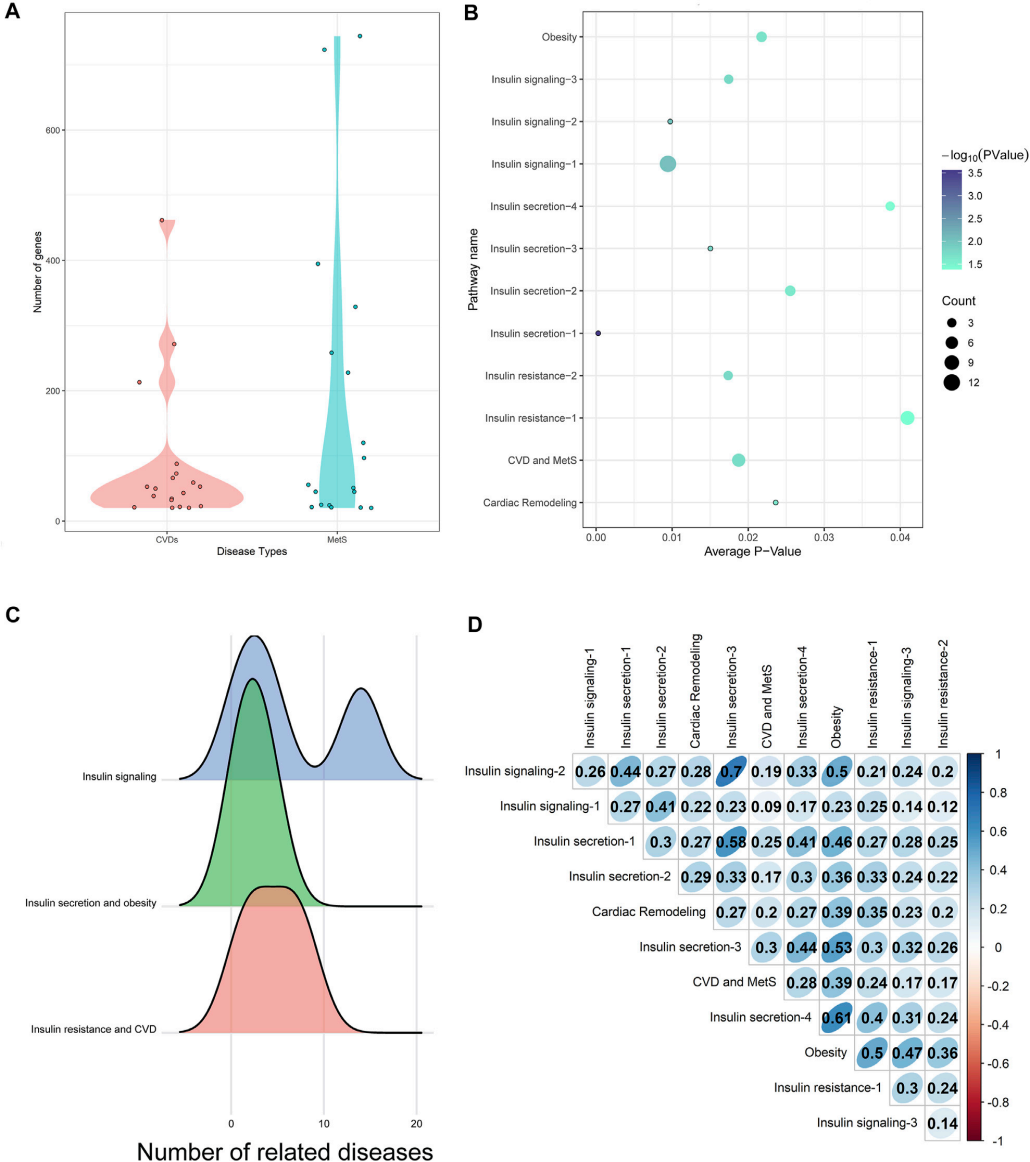
**(A)** Distribution of genes in CVDs and MetS. **(B)** Common pathways enriched by HM datasets. The diameter of each bubble represented the number of diseases significantly enriched in this pathway. **(C)** Relationships between common pathways and diseases. **(D)** Network similarity of common pathways.

CVD (such as coronary disease) and MetS (such as diabetes mellitus) lists were extracted from Medical Subject Heading (MeSH) ontology with at least 20 disease-related genes from either OMIM or GWAS (listed in Supplementary Table S1). 553 disease pairs were shown to be similar with each other with a *z*-score ≥ 1.6 and *q*-value ≤ 0.001. The 19 CVDs and 18 MetS comprising the 553 disease pairs were selected as HM (HeartMetS) datasets. As shown in Figure 2A, the average numbers of genes related to MetS (179.0556) were 2-fold of CVDs (86.42105). This indicated that MetS may be more complex than CVDs since these diseases involve the abnormality of multiple systems, such as endocrine, digestive, and immune systems.

The gene lists of each disease were then used as inputs of the information converting calculation. 179 pathways in KEGG (Kanehisa et al., 2017) and Biocarta with at least one *e*-value not above 0.05 were selected as HM-enriched pathways (see Supplementary Table S2). The common pathways in the two databases were named using KEGG ID. Otherwise, if there exists any difference between the two pathways, both of the pathways were kept.

CA was shown to play important roles through biological pathways in reducing metabolic syndrome complications and CVDs as reviewed in the former research (Yang et al., 2015; Mollazadeh and Hosseinzadeh, 2016). The words “cinnamon” and “cinnamaldehyde” were used for literature searching through the NCBI PubMed to find the related pathways since 32 CA-related pathways were selected and binned into two groups according to their effects on diseases types: antidiabetic (including 28 pathways) and antihypertensive (including four pathways).

### Combined Pathway Network Analyses

As shown in Figure 2B, there were 12 common pathways between the 179 HM-enriched pathways and 32 CA-related pathways. This indicated the dynamical roles CA played in different diseases or disease stages, including diabetes mellitus, obesity, MetS, and CVDs. The combined pathway network was built using the 12 common pathways as nodes. Sixty-seven connections were built if the two pathways shared at least one gene/protein.

The 12 common pathways were enriched by different numbers of diseases in the HM datasets. Of which, the insulin signaling pathway (hsa04910, marked as “Insulin signaling-1”) was enriched by 12 diseases, while the following three pathways were only enriched by one disease: IL-2 receptor beta chain in T-cell activation (h_il2rbPathway, marked as “Cardiac Remodeling”), the IGF-1 receptor and longevity (h_longevityPathway, marked as “Insulin secretion-1”), multiple antiapoptotic pathways from IGF-1r signaling lead to bad phosphorylation (h_igf1rPathway, marked as “Insulin secretion-3”), and sprouty regulation of tyrosine kinase signals (hsa04911, marked as “Insulin signaling-2”).

The 12 common pathways could be divided into three types according to their contributions to CVD and MetS (as shown in Figure 2C).

1) Insulin signaling: CA could enhance the insulin signaling pathway in the skeletal muscle by increasing the tyrosine phosphorylation level (Qin et al., 2003). Three pathways were involved in this stage, including the insulin signaling pathway (enriched by MetS and CVDs), tyrosine metabolism (enriched by MetS), and sprout regulation of tyrosine kinase signals (enriched by CVD). It was interesting to see that the “insulin signaling pathway” was closely connected not only to MetS such as diabetes mellitus but also to CVDs, such as heart diseases. This may be explained by the fact that insulin signaling was an integral pathway regulating the life span of laboratory organisms (Schriner et al., 2014).
2) Insulin secretion and obesity: Since impaired insulin secretion was one of the pathophysiological abnormalities in type 2 diabetes, IGF (insulin-like growth factors)-I, which was shown to inhibit insulin secretion, would play a key role in the process (Leahy and Vandekerkhove, 1990; Pørksen et al., 1997). CA could increase the phosphorylation levels of the IGF-I receptor and its downstream signaling molecules (Takasao et al., 2012). It was interesting that binding IGF-I to its receptor could cause the activation of the tyrosine kinase, leading to autophosphorylation of the intrinsic tyrosines, which transduced the IGF-I signal to a complex network that was ultimately responsible for cell proliferation, modulation of tissue differentiation, and protection from apoptosis (Laviola et al., 2007).
3) Insulin resistance and CVD: The study showed that the insulin action on cAMP was severely impaired in insulin-resistant patients (Laviola et al., 2007). The cyclic-AMP signaling pathway was shown to be modulated by CA to exhibit antidiabetic action (Schriner et al., 2014). “Regulation of lipolysis in adipocytes” (marked as “Obesity”) was closely linked to MetS since the variations of insulin resistance severity may be related to the regulation of lipolysis in adipocytes (Guilherme et al., 2008). The “AMPK signaling pathway” was proven to be a master regulator of key molecular effectors involved in both metabolic processes and cardiovascular homeostasis by modulating the mTOR signaling and IGF-1 pathway (Salminen and Kaarniranta, 2012). The pathway “Il-2 receptor beta chain in T-cell activation” was proven to significantly attenuate ventricular remodeling by reducing infarct size and improving left ventricular (LV) function (Zeng et al., 2016).

The *S*_*GDD*_ and *S*_*AE*_ were calculated for all the 32 CA-related pathways and the 179 HM-enriched pathways. Overall, the average intra-similarity (pathways of the same types including “insulin signaling,” “insulin secretion and obesity,” and “insulin resistance and CVD” as illustrated above) in either CA-related or HM-enriched pathways was similar: higher *S*_*GDD*_ score and lower *S*_*AE*_ scores (see Table 1 for details). This indicated that these pathways may have small similar structures instead of the whole network. Each pathway may be an up or downstream event in a disease since the biological processes inducing diseases were complex. There may be local similar structures between two pathways, especially the adjacent ones, that may help transform the information quickly.

**TABLE 1:** Pathway similarity results of different pathways.

Type of pathway sets	Number of pathways	*S*_*GDD*_	*S*_*AE*_
Common	12	0.257095099	0.047247541
CA-related	32	0.355600743	0.091677581
HM-related	179	0.324632623	0.061221968

The combined pathway network similarity scores between the 12 common pathways are shown in Figure 2D. Of which, the pathway “Regulation of lipolysis in adipocytes (hsa04923)” (marked as “Obesity” in Figure 2D) got the highest average combined network similarity score (0.438) in the 12 common pathways. As illustrated above, this pathway was involved in the “Insulin resistance and CVD” processes of CVD and MetS, which was the downstream event of CVD and MetS, indicating that more cross-talks may exist between this pathway and the upstream events through the similarity network structures. Compared with this, the pathway “Insulin signaling pathway (hsa04910/h_insulinPathway)” (marked as “Insulin signaling-1” in Figure 2D) got the smallest average combined network similarity score (0.218). Interestingly, this pathway was the node with the highest degree 15 in the combined pathway network. Considering the biological character of this pathway, these indicated that this upstream event in MetS and CVD may play a triggering role regardless of structure similarities to other downstream pathways.

### Peptide–Protein–Based Drug Targets Selection

The combined protein network was built using all the proteins of the 12 common pathways. The network comprised 335 nodes and 1793 edges. The proteins with top 10 degree, node betweenness, and edge betweenness are listed in Table 2 and selected as raw candidate biomarkers. The degree of a node indicated the importance of a node in the network. A higher degree meant more connections with other nodes; thus, the proteins with higher degree may be the key targets of CA. Five of the top 10 degree proteins had been proven to be regulated by CA, including IRS1, AMPK1, AMPK2, PRKAB1, and PRKAB2. The other five proteins could be divided into two groups: monoamine oxidase (MAOA and MAOB) and protein kinase AMP-activated non-catalytic subunit gamma (PRKAG1, PRKAG2, and PRKAG3) which may be the candidate targets of CA. Cinnamon extracts (CEs) were shown to increase insulin sensitivity by increasing the mRNA expression of INSR (insulin receptor) (Anderson et al., 2013), promoting IRS1 (insulin receptor substrate 1) phosphorylation (Liu et al., 2016), and activating AMPK1/2 (protein kinase AMP-activated catalytic subunit alpha 1/2) (Hu et al., 2013). On the contrary, CE was shown to decrease the expression of genes encoding insulin signaling pathway proteins, including IGF1R (Cao et al., 2010). INS-encoded insulin and trimer procyanidins in CE were shown to contribute to the INS-1 pancreatic β-cell protection (Sun et al., 2016).

**TABLE 2:** List of top 10 nodes and edges in the combined protein network.

Topological character	Protein symbols/protein–protein pairs
Degree	IRS1, MAOA, MAOB, AMPK1, AMPK2, PRKAB1, PRKAB2, PRKAG1, PRKAG2, and PRKAG3
Node betweenness	IRS1, OGT, AKT2, INS, AKT1, RAPGEF4, INSR, PDE3B, PTPA, and GNAS
Edge betweenness	PTPA-AKT2, IRS1-IGF1R, PPARGC1A-OGT, AKT1-E2F1, IGF1R-RAF1, AKT2-PDE3B, OGT-AKT1, E2F1-IL2RA, PRKCE-INSR, and NOS3-IRS1

Compared with degree, the measure “betweenness” reflected the importance of proteins/protein–protein pairs in the interplays between different pathways/diseases. Three of the top 10 betweenness proteins, including IRS1, INS, and INSR, were validated to be regulated by CA. Six of the 10 betweenness edges contained at least one validated CA target. It was found that the two nodes forming the edge IRS1-IGF1R were CA targets; however, IRS1 was upregulated, while IGF1R was downregulated, indicating there may exist complex interactions between CA targets.

**FIGURE 3:**
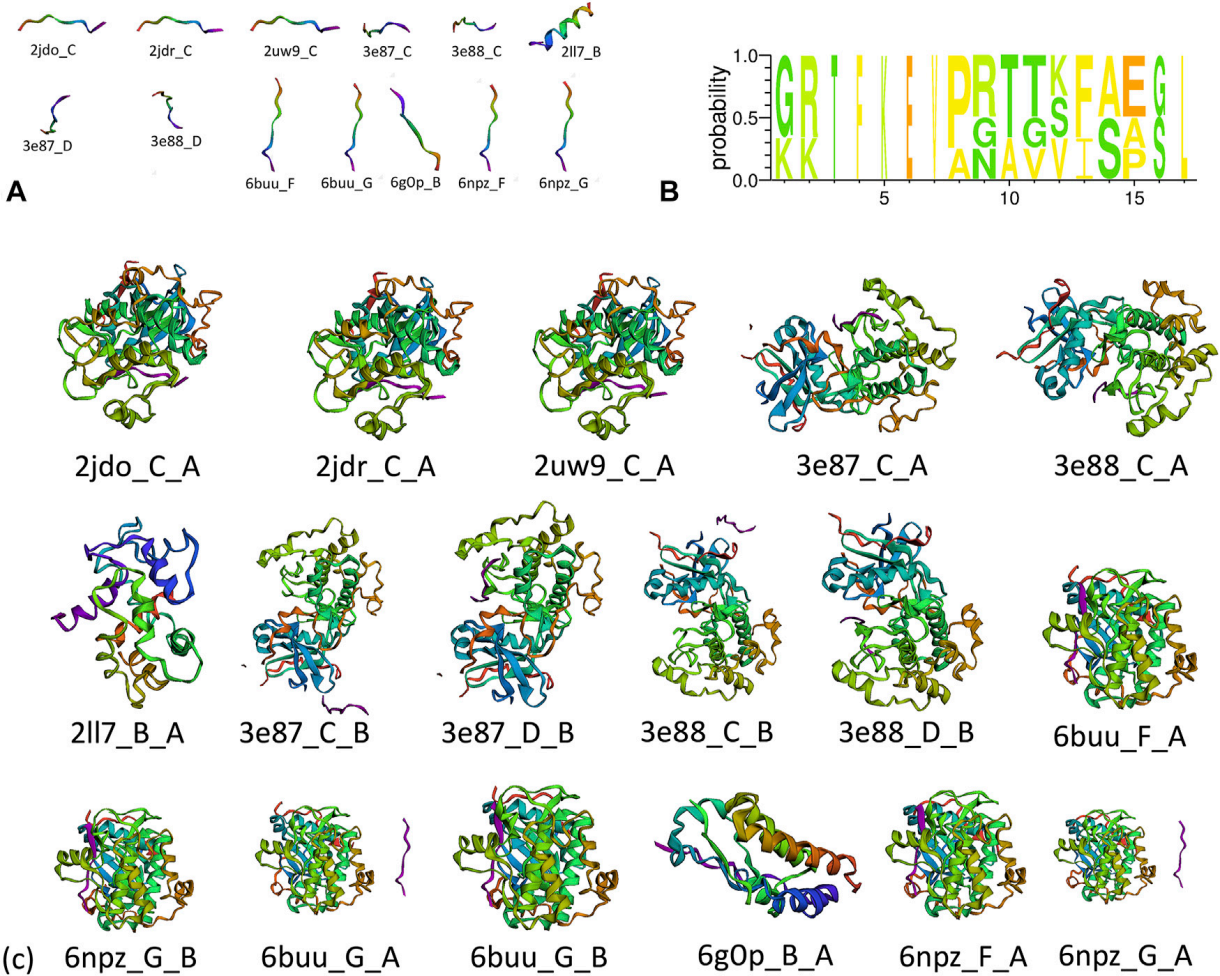
**(A)** Structure of clustered peptides, **(B)** sequence alignment of clustered peptides, and **(c)** structure of filtered peptide–protein complexes.

**FIGURE 4:**
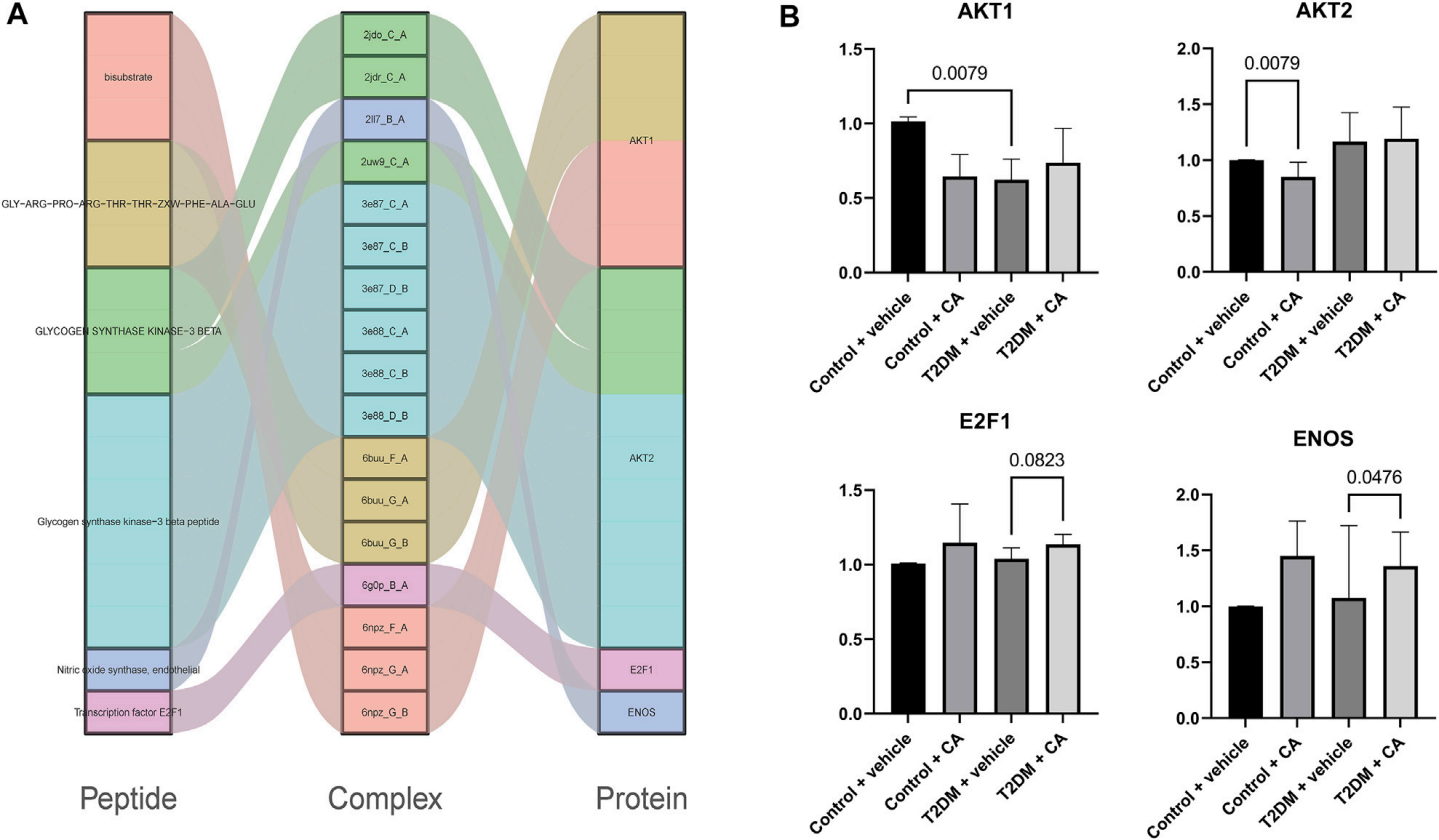
**(A)** Relationships between peptides and proteins, and **(B)** qRT-PCR results of AKT1, AKT2, E2F1, and ENOS.

A total of 67 protein–peptide complexes containing these top proteins as receptors were selected, and the peptides in these were then aligned and clustered. In total, 13 peptides were grouped in the CA-related cluster, their characters are listed in Table 3, and the structures are shown in Figure 3A,B. In total, 17 peptide–protein complexes were then filtered (see Figure 3C for the complexes’ structures), see Figure 4A for the relationships between these peptides and proteins.

**TABLE 3:** Peptide in the CA-related cluster.

PDB	Peptide chain	Peptide size	Peptide sequence	Peptide description	Peptide molecular weight	Peptide aromaticity	Peptide instability	Peptide isoelectric point
6buu	F	11	GRPRTTXFAEX	GLY-ARG-PRO-ARG-THR-THR-ZXW-PHE-ALA-GLU	−	0.09	−	9.6
6buu	G	11	GRPRTTXFAEX	GLY-ARG-PRO-ARG-THR-THR-ZXW-PHE-ALA-GLU	−	0.09	−	9.6
6npz	F	11	GRPRTTXFAEX	Bisubstrate	−	0.09	−	9.6
6npz	G	11	GRPRTTXFAEX	Bisubstrate	−	0.09	−	9.6
2jdo	C	10	GRPRTTSFAE	Glycogen synthase kinase-3 beta	1121.2	0.1	20.72	9.6
2jdr	C	10	GRPRTTSFAE	Glycogen synthase kinase-3 beta	1121.2	0.1	20.72	9.6
2uw9	C	10	GRPRTTSFAE	Glycogen synthase kinase-3 beta	1121.2	0.1	20.72	9.6
3e87	C	10	GRPRTTSFAE	Glycogen synthase kinase-3 beta peptide	1121.2	0.1	20.72	9.6
3e87	D	10	GRPRTTSFAE	Glycogen synthase kinase-3 beta peptide	1121.2	0.1	20.72	9.6
3e88	C	10	GRPRTTSFAE	Glycogen synthase kinase-3 beta peptide	1121.2	0.1	20.72	9.6
3e88	D	10	GRPRTTSFAE	Glycogen synthase kinase-3 beta peptide	1121.2	0.1	20.72	9.6
6g0p	B	9	PGXGVXSPG	Transcription factor E2F1	-	0	-	5.96
2ll7	B	17	KKTFKEVANAVKISASL	Nitric oxide synthase, endothelial	1834.16	0.06	1.14	10

Four of the raw candidate biomarkers (AKT1, AKT2, E2F1, and ENOS) were receptors of the abovementioned 17 peptide–protein complexes*.* qRT-PCR was performed on the four genes (see Supplementary Table S2 for details).

The candidate biomarkers were divided into three groups according to their expression changing pattern in the qRT-PCR results as follows: see Figure 4B for details 1) The genes differentially expressed between Group A (Control + vehicle) and Group B (Control + CA) were named as CA-indicating biomarkers since the two groups were under normal condition, while the only difference between the two groups was the drug CA. 2) The genes differentially expressed between Group A (Control + vehicle) and Group C (T2D + vehicle) were named as T2D responsive biomarkers since these genes were significantly differentially expressed between T2DM and controls but were not related to the drug CA. 3) The genes differentially expressed between Group C (T2D + vehicle) and Group D (T2D + CA) were named as CA treatment targets since the samples of the two groups were all T2D, while the only difference between them was the treatment of CA. Of which, AKT2 was a CA-indicating biomarker and AKT1 was a T2D responsive biomarker, while E2F1 and ENOS were CA treatment targets. E2F1 and ENOS were shown to cooperate with each other in the treatment of hypertension (Li et al., 2019). Combined with results from this study, the two genes might also cooperate with each other in T2D and become the targets of CA. Besides, the two genes were found to be targeted by SARS-CoV-2–encoded miRNAs in recent research (Aydemir et al., 2021). As a result, CA may be a potential candidate drug to help reduce or prevent the complications since CVDs were one of the most common complications in COVID-19 patients.

## Discussion

The analysis pipeline that was proposed in this study was based on the related genes of multiple diseases. In this study, these genes were collected from OMIM and GWAS results; however, the updates of the gene lists might only influence the results slightly since the analyses were performed on pathway levels. The information conversion from genes to pathways could capture most of the functional characters of the disease, which may not be changed by adding or deleting a small number of genes. CA was shown to play roles in a wide disease spectrum, which was the character of many Chinese traditional medicines. Thus, the drug targets of these diseases may share some similar characters reflected by peptide clusters. The pipeline proposed in this study could be applied to other diseases and drugs. Pathways were commonly used in biological and medical analyses which could gain deep understanding of diseases. However, other biological terms that could be converted into networks could also be used in this pipeline.

The portability of the pipeline was shown in all the three steps. In step 1 (pathway filter), the similarity calculation methods between different disease pairs could be replaced by any suitable distance measures. The disease-related and drug-related pathways could be selected using any suitable scores or ways. Other functional resources and transcriptional information such as GO terms, transcriptional factors–targets, or miRNA targets could also be used. However, pathways were recommended as the primary choice because the biological pathways were widely used in biological and medical analyses since they could reflect the molecular connections in the form of graphs, which could be analyzed using multiple computational methods. Besides, the correlations between pathways and peptides were closer than those between other types of functional resources. In step 2 (combined network construction), the network structure similarities could be measured using one alignment-free and one alignment-based algorithms. In step 3 (biomarker prediction and validation based on peptides), the peptide clustering algorithms could be replaced by any other suitable alignment method.

## Conclusion

In this study, a new pipeline was proposed to discover drug targets based on peptides. The network analyses based on machine learning methods could quantify the effects of peptide–protein complexes with similar structures as drug targets in multiple diseases.

## Data Availability

The original contributions presented in the study are included in the article/Supplementary Material; further inquiries can be directed to the corresponding authors.
